# Precision Landing of a Quadcopter Drone by Smartphone Video Guidance Sensor in a GPS-Denied Environment

**DOI:** 10.3390/s23041934

**Published:** 2023-02-09

**Authors:** Nicolas Bautista, Hector Gutierrez, John Inness, John Rakoczy

**Affiliations:** 1 Department of Mechanical and Aerospace Engineering, Florida Institute of Technology, Melbourne, FL 32901, USA; 2 Control Systems Design and Analysis Branch, NASA Marshall Space Flight Center, Huntsville, AL 35812, USA

**Keywords:** unmanned aerial vehicle, autonomous landing, smartphone video guidance sensor, visual inertial odometry, GPS-denied environment

## Abstract

This paper describes the deployment, integration, and demonstration of a Smartphone Video Guidance Sensor (SVGS) as a novel technology for autonomous 6-DOF proximity maneuvers and precision landing of a quadcopter drone. The proposed approach uses a vision-based photogrammetric position and attitude sensor (SVGS) to estimate the position of a landing target after video capture. A visual inertial odometry sensor (VIO) is used to provide position estimates of the UAV in a ground coordinate system during flight on a GPS-denied environment. The integration of both SVGS and VIO sensors enables the accurate updating of position setpoints during landing, providing improved performance compared with VIO-only landing, as shown in landing experiments. The proposed technique also shows significant operational advantages compared with state-of-the-art sensors for indoor landing, such as those based on augmented reality (AR) markers.

## 1. Introduction

The use of automated unmanned aerial vehicles (UAVs) has significantly increased in recent years due to their portability, low cost, and high performance. Multi-copters have become a leading platform for research and development in automated flight due to their versatility and ease of development. The navigation of UAVs is significantly different in outdoor scenarios, where the flight controller relies on GPS to estimate position, from indoor missions, where position estimates need to be based on other sensors because GPS signal is not available.

A leading approach for indoor automated precision landing has been based on the image capture of AR (augmented reality) markers, where images captured during flight are processed using image recognition algorithms, such as those available in OpenCV. A second important approach in the state of the art is based on the use of an infrared landing beacon to estimate the distance to the landing target and provide guidance commands, but in practical terms, this approach is restricted to outdoor landing scenarios.

Zhao and Jiang [1], Tanaka and Matsumoto [2], Sani and Karimian [3], and Wang et al. [4] have presented algorithms to generate unique black and white AR markers. Tanaka [2] also replaced a square of their AR marker with a lenticular angle gauge to improve attitude estimates. Putra et al. [5], Respall et al. [6], and Tran et al. [7] used color detection algorithms to estimate the position of the center of a solid color shape on a contrasting background. Sudevan et al. [8] and Demirhan and Permachandra [9] used a letter “H” stamped on their ground target for their system to detect the contours of the letter and estimate landing position. Rao et al. [10] used a circle with a different color in each half, and concentric rectangles inside the circle as markers. The studies mentioned above used image detection algorithms to determine the position of the landing marker relative to the drone during flight, and the estimate was then used to send velocity commands to the flight controller of the UAV. One of the main drawbacks of these methods is that a well-lit target is needed to properly estimate position and attitude: such methods cannot work well in dusty or poorly lit environments, such as in planetary landing. There are also range limitations in the use of AR targets imposed by target size and camera resolution.

Xuan-Mung et al. [11] and Janousek and Marcon [12] described the use of infrared (IR) beacons to estimate distance to a landing target, using a camera with an infrared filter. This method can be advantageous in applications with poor or no lighting, because the camera is able to detect the beacon regardless of lighting conditions. The main drawback of this method is given by false readings that can be generated by the reflection of sun rays on certain surfaces, and possibly certain heat sources, and the fact that it generally requires a detection distance of more than 10 m.

In all references mentioned above, the vehicles included a sensor to detect the landing target. Kim et al. [13] used a 3D lidar sensor mounted on the landing platform. Their landing method approaches the target using known GPS coordinates, and once the lidar is able to detect the position of the UAV, velocity commands are sent to the drone’s flight controller wirelessly. This method lowers the payload weight of the UAV, because hardware components needed for position estimation of the landing target are no longer needed in the flight platform. The approach also requires a heavier, complex landing platform, which might not be possible in certain missions.

Several approaches for automated landing have been based on camera-based feedback, such as studies by Ho and Chu [14], Demirhan [9], and Sikdar [15]. These approaches all share the limitations of requiring favorable light conditions, and are therefore not well suited for poorly lit or dusty environments. Other vision-based approaches address these issues using LED landing strips (Bastiaens et. al. [16]), or by the combined use of AR markers and infrared sensing (Badakis et. al. [17]), which improves performance at the expense of added complexity.

This paper proposes a solution to achieve precise autonomous landing in a GPS-denied environment using the Smartphone Video Guidance Sensor (SVGS) [18,19], a software sensor developed by NASA that uses a smartphone’s CPU and camera to detect the position and orientation of a landing target. In the proposed solution, SVGS is integrated with a Visual Inertial Odometry (VIO) sensor to estimate the UAV’s position and attitude during flight in a GPS-denied environment.

## 2. Materials and Methods

SVGS [18,19] uses an illuminated target and an Android smartphone running the SVGS application. SVGS uses the smartphone’s camera to capture images of the illuminated target and estimates the 6-DOF position and attitude of the target relative to the phone’s coordinate system using a photogrammetric algorithm.

The approach described in this paper uses a visual inertial odometry (VIO) tracking camera to estimate the 6-DOF state of the drone relative to a fixed coordinate system attached to the ground. The use of VIO is required in indoor missions because the usual source of position estimates to the drone’s FCU is the onboard GPS sensor, and GPS signals are not available in indoor environments. Using the VIO sensor in combination with SVGS, it is possible to achieve accurate estimation of the position of the landing target in a coordinate system fixed to the ground. This enables the use of updated position setpoints during the landing mission, which works even if the SVGS device loses line of sight with the target during the landing maneuver. The performance of SVGS as a landing sensor will be compared with that obtained using AR markers for automated landing [20,21]. ARuCo defines a modified AR target based on an OpenCV approach, and was used as a special case of AR landing target.

### 2.1. Smartphone Video Guidance Sensor

**Figure 1:**
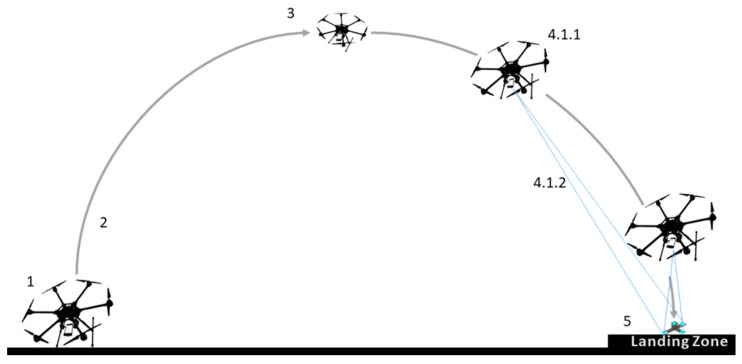
Drone landing mission using the Smartphone Video Guidance Sensor (SVGS). The mission sequence numbers correspond to the landing logic shown in Figure 2. The SVGS can be deployed in many other applications as position/attitude sensor by use of a similar 4-point illuminated beacon [22].

The Smartphone Video Guidance Sensor (SVGS) is a low-cost, low-mass, vision-based sensor developed by NASA Marshall Space Flight Center [18,19] that computes the six-state position and orientation vector of a target relative to a coordinate system attached to the camera of an Android-based smartphone, by capturing images and implementing photogrammetric techniques. Figure 1 outlines the operational concept of a drone landing mission using SVGS. SVGS operation starts with image capture of the illuminated target, which is then converted to a binary image, so that the four bright spots on the target can be identified as blobs. Estimation of the six-degrees-of-freedom position and attitude state vector is then performed using a photogrammetric algorithm [18]. The smartphone’s CPU is used to perform both image processing and state estimation, thus alleviating the computational load on the flight or companion computer [18].

#### SVGS Theory of Operation

The SVGS algorithm as shown in [18] is based on an adaption of the collinearity equations developed by Rakoczy [23]. In Figure 3, consider L as the perspective center of the host platform camera (thin lens), and the target as A. An image of A on the image plane is represented as “a”; the image plane is located a distance, f, from the perspective center, where f is the focal length of the lens. Two coordinate frames are defined: the target frame <*X, Y, Z*>, and the image frame <*x, y, z*>. A vector from the perspective center to point “*A*” can be defined in the object frame as *V_A_*, while a vector to point “a” from the perspective center is *v_A_*:

(1) $$v_{A}=\left[\begin{array}{c} X_{A}-X_{L}\\ Y_{A}-Y_{L}\\ Z_{A}-Z_{L} \end{array}\right]\begin{array}{ccc} & ; & \end{array}v_{a}=\left[\begin{array}{c} x_{a}-x_{0}\\ y_{a}-y_{0}\\ -f \end{array}\right]$$

Both vectors are related by $v_{a}=kMv_{A}$, where *k* is a scaling factor and *M* is a rotation matrix representing an x, y, z rotation sequence transforming the object frame to the image frame. Dropping the “*a*” and “*A*” subscripts and solving for the image frame coordinates *x*, *y*, and *z* of point “a”, followed by dividing by “*z*” to eliminate the scaling factor *k*, yields the following two equations for *F_x_* and *F_y_*, where the *m_ij_* values are elements of the direction cosine matrix *M*:

(2) $$x=f\frac{m_{11}\left(X-X_{L}\right)+m_{12}\left(Y-Y_{L}\right)+m_{13}\left(Z-Z_{L}\right)}{m_{31}\left(X-X_{L}\right)+m_{32}\left(Y-Y_{L}\right)+m_{33}\left(Z-Z_{L}\right)}+x_{0}=F_{x}$$

(3) $$y=f\frac{m_{21}\left(X-X_{L}\right)+m_{22}\left(Y-Y_{L}\right)+m_{23}\left(Z-Z_{L}\right)}{m_{31}\left(X-X_{L}\right)+m_{32}\left(Y-Y_{L}\right)+m_{33}\left(Z-Z_{L}\right)}+y_{0}=F_{y}$$

The relative 6-DOF state vector that needs to be solved for is *V* is defined below, where *φ*, *θ*, and *ψ* represent the *x*, *y*, and *z* rotation angles, respectively:

(4) $$V={\left[\begin{array}{cccccc} X_{L} & Y_{L} & Z_{L} & \phi & \theta & \psi \end{array}\right]}^{T}$$

Linearizing *F_x_* and *F_y_* by Taylor series truncated after the second term yields:

(5) $$x=F_{x}(V_{0})+\frac{\partial F_{x}}{\partial V}\Delta V+\varepsilon_{x},\;y=F_{y}(V_{0})+\frac{\partial F_{y}}{\partial V}\Delta V+\varepsilon_{y}$$

where *V*_0_ is an initial guess for the state vector, Δ*V* is the difference between this guess and the actual state vector $\Delta V=V-V_{0}$, and *ε_x_* and *ε_y_* are the *x* and *y* error from the Taylor series approximation, respectively. Each of the four targets in the SVGS target pattern has a corresponding set of these two equations; the resulting eight equations can be represented in matrix form as $Y=Y_{0}+HV+\varepsilon$:

(6) $$Y=[\begin{array}{c} x_{1}\\ y_{1}\\ ..\\ ..\\ x_{4}\\ y_{4} \end{array}];\;\;\;Y_{0}=[\begin{array}{c} F_{x_{1}}\\ F_{y_{1}}\\ ..\\ ..\\ F_{x_{4}}\\ F_{y_{4}} \end{array}];\;\;\;H={\left[\begin{array}{cccccc} \frac{\partial F_{x_{1}}}{\partial V} & \frac{\partial F_{y_{1}}}{\partial V} & .. & .. & \frac{\partial F_{x_{4}}}{\partial V} & \frac{\partial F_{y_{4}}}{\partial V} \end{array}\right]}^{T}$$

This equation is solved for the *V* that minimizes the square of the residuals, ε. This value is then added to the initial estimate of *V* to obtain the updated state vector. The process is iterated until the residuals are sufficiently small, yielding the final estimate of the 6-DOF state vector *V*.

In SVGS, the general form of the collinearity equations described above is narrowed down to reflect the state vector formulation used by AVGS [19]. AVGS sensor measurements used angle pairs, azimuth, and elevation, measured in the image frame to define the location of each target in the image. Azimuth and elevation are measured with respect to a vector to the perspective center and the target locations in the captured image. Azimuth, *A_z_*, and elevation, *E_l_* (Equation (7)), replace Equations (2) and (3) to yield:

(7) $$A_{z}=\tan^{-1}\left(\frac{x-x_{0}}{f}\right);\;E_{l}=\sin^{-1}\left(\frac{y-y_{0}}{\sqrt{{\left(x-x_{0}\right)}^{2}+{\left(y-y_{0}\right)}^{2}+f^{2}}}\right)$$

The SVGS calculation begins with the capture of the illuminated pattern on the target. The image is then converted to a binary image using a specified brightness threshold value. Blob extraction is performed on the binary image to find all bright spot locations. Location, size, and shape characteristics of the blobs are captured. Depending on whether there are any other objects in the field of view that may generate bright background-noise spots, the number of blobs may exceed the number of targets. To account for noise and properly identify which target is which, a subset of four blobs is selected from among all that are identified, and geometric alignment checks derived from the known orientation of the targets are applied. The process is iterated until the four targets have been identified and properly labeled. The target centroids are then fed into the state determination algorithms. Using the collinearity formulation (Equation (7)), the relative state is determined using a least-squares procedure. The SVGS algorithm flow is shown in Figure 4 [18,19].

### 2.2. Description of the Flight Platform

The landing experiments used a quadcopter based on the Holybro X500 frame (Holybro Ltd., Shen Zhen, China) (Figure 5); Figure 6 shows the corresponding interconnection diagram.

The drone system included: (i) a Pixhawk-4 flight controller (FCU) running PX4 firmware; (ii) a LattePanda Alpha 864 companion computer (64-bit Intel m3-8100Y CPU with 8 GB LPDDR3 RAM) running Ubuntu, as companion computer; (iii) a Samsung Galaxy S8, used as the SVGS device; (iv) an Intel T265 tracking camera, used as Visual Inertial Odometry (VIO) sensor; (v) a IOIO board, for serial communication between the SVGS smartphone and companion computer, and a four-cell lithium-ion polymer battery to power the system. The quadcopter included four DC brushless motors and four electronic speed controllers (ESCs) powered from a power distribution board connected to the battery. The ESCs receive signal from a PWM splitter connected to the FCU, which communicates with a ground station through a telemetry radio link, and with the companion computer through a second serial telemetry port using MAVLink (FCU communication protocol).

The companion computer receives timestamped odometry data from the Intel T265 Visual Inertial Odometry sensor (VIO) through a 3.0 USB connection, and serial SVGS data via a USB to UART connector from the IOIO board, connected to the SVGS device (Samsung Galaxy 8, Samsung, Suwon-Si, Korea) via Bluetooth. In the experiments using ArUco, the SVGS device and the IOIO board are removed, and are replaced with a webcam connected to the companion computer via USB. The landing targets for SVGS and ArUco are shown in Figure 7. For SVGS, an illuminated LED target was used; for the AR landing, an ArUco marker was used in combination with a USB webcam.

### 2.3. Overview of Landing Mission

**Figure 2:**
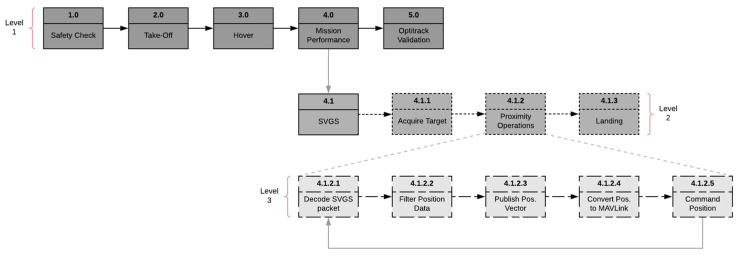
SVGS landing mission—functional flow diagram.

**Figure 3:**
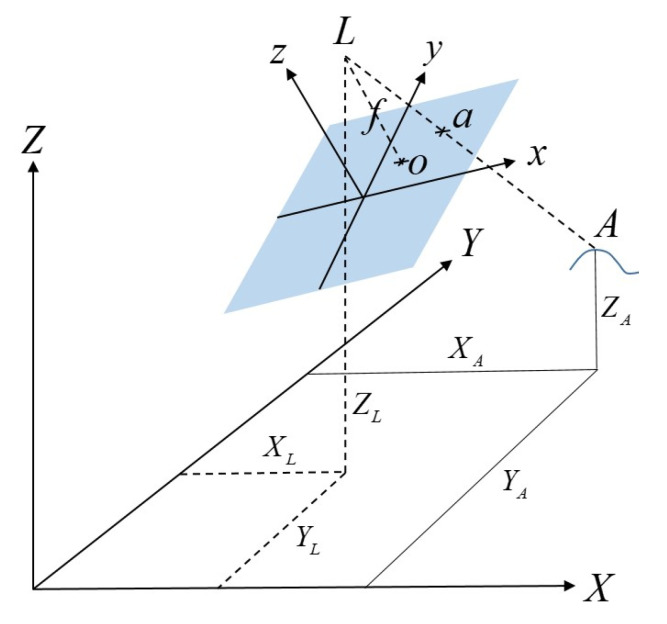
Object and camera frame geometry of SVGS. The image plane is shown in blue.

**Figure 4:**
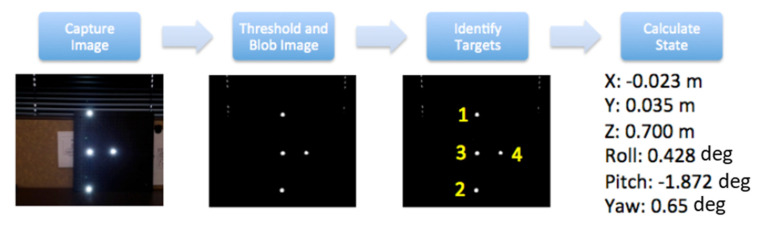
SVGS algorithm flow [18,19], and example output vector.

**Figure 5:**
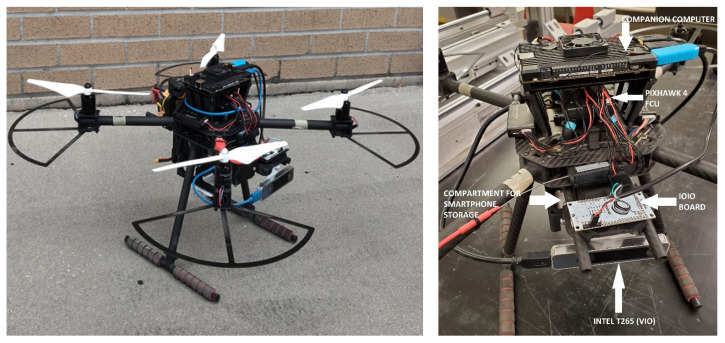
Flight platform used in the development and testing of landing experiments.

**Figure 6:**
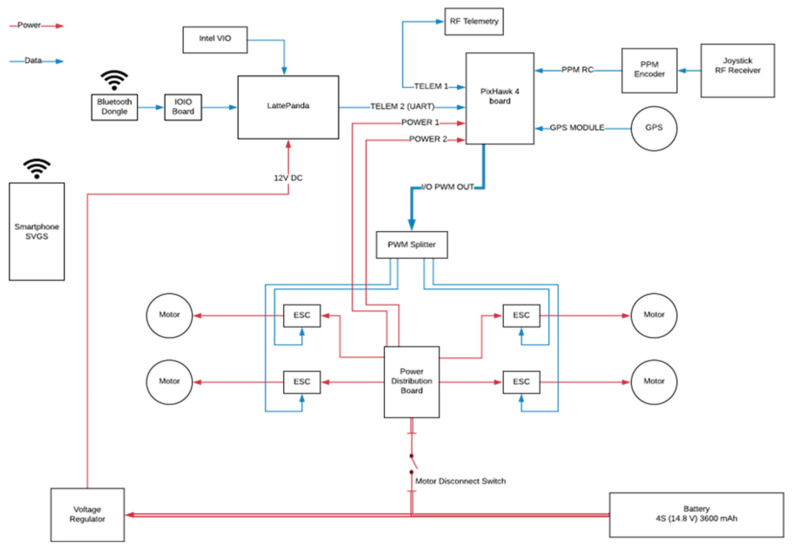
Interconnection diagram of the quadcopter flight platform.

Landing missions start with a preflight safety check, as shown in Figure 2. Take-off and approach to the landing target are controlled manually; the pilot can switch to autonomous mode at any time using a manual switch integrated with the joystick controller. After a hovering period to stabilize the drone trajectory, the drone acquires the landing target and estimates its position and attitude in the coordinate frame fixed to the ground using SVGS readings and the VIO sensor estimate. After the target is acquired, the automated landing portion of the mission is shown in block 4.1.2. The software for automated landing was developed in the companion computer in ROS/Python, as described in Section 2.4.

The autonomous landing logic is shown in Figure 8. The target position is saved in the memory and updated every time a reading is captured: if the target is lost from the camera view during landing, the latest valid target position estimate saved in the memory is used. After target lock in SVGS is achieved, (X,Y) position setpoints are sent to the FCU based on the current SVGS reading, directing it to the estimated landing target while maintaining a constant altitude. Once the drone is within an acceptable radius of the target location (in the horizontal plane) a timer counts to a specified number of seconds, and if the UAV location leaves the acceptable radius, the timer starts over. This ensures that a stable position above the target is achieved before descent. When the timer reaches the specified value (i.e., the vehicle has remained within tolerance above the landing target for sufficient time), the drone descends at constant vertical speed. Figure 8 shows the flowchart for the autonomous portion of the mission.

### 2.4. Software Development and Filtering

Flight software in the companion computer was developed in a robot operating system (ROS). ROS consists of a set of communication and scheduling interfaces that use a publisher/subscriber communications architecture and are widely used in robotics applications. They enable to discretization of the required software functionality into software packages called nodes, which are easily modified and reused to support different tasks. The PX4 flight stack has extensive integration with ROSs, particularly through the MAVROS package. This enables the straightforward integration of several sensors, enabling the development of complex systems.

ROS works based on packages; a package is a set of programs called nodes. Nodes pass data, called topics, over specified ports to each other; ROS Kinetic with Ubuntu 16.04 was used in the companion computer. ROSs allow communication between packages; therefore, two open-source ROS packages were used: MAVROS, to support communication with the FCU using MAVLink via UART connection over USB (FCU communication protocol and serial interpreter), and px4_realsense_bridge, to fuse the Intel VIO sensor estimate with the vehicle’s IMU readings. To communicate SVGS data to the FCU, a new ROS package called “DR1” was created using Python nodes that communicate with the MAVROS and px4_realsense_bridge packages. Figure 9 illustrates how ROS nodes communicate.

The nodes created for DR1 are:

“**svgs_deserializer**”: converts serial data packets from SVGS and publishes the output as a stamped twist message on the topic “target”, with a data type of twist (a data structure used by ROSs that includes a timestamped translational and angular coordinates).

“**aruco_state_estimator**”: performs the same task as svgs_deserializer, except it uses a Logitech C920 webcam and the ArUco target to generate the estimates.

“**landing_commander**”: checks that the current drone position relative to target and magnitude of the drone velocity are within their acceptable threshold values. If they are, the node starts the landing counter. Once the counter hits a threshold value, the node flips the landing flag to true, which commands the drone to start its descent.

“**motion_control**”: this node depends on the landing flag from the “landing_commander” node. If the landing flag is false, the node updates the setpoint to be the position of the target (from the “svgs_deserializer” node) while maintaining its current altitude. Once the landing flag is true (landing criteria is met), it updates the setpoint to be the position of the landing target.

“**px4_control**”: this node subscribes to the “motion_control” node. It obtains setpoint data and sends the setpoint data to the FCU at 30 updates/sec.

“**data_recording**”: subscribes to variables and records them in a CSV file at 25 Hz.

The SVGS is not a real-time sensor and has a high degree of variability on its update rate [14]. SVGS data are non-deterministic for two reasons: the SVGS runs on an Android-based system, and SVGS data are processed in the Ubuntu-based companion computer. Due to non-deterministic timing inherent to any operating system, SVGS samples are not available at constant sampling rates, but instead follow a certain statistical distribution [14]. For this reason, attempting to estimate velocity using the filtered finite-differences of the SVGS position estimate leads to highly noisy and inaccurate estimates. The proposed solution for SVGS velocity estimation is based on a second-order kinematic Kalman filter:

(8) $$x_{k+1}=x_{k}+\dot{x_{k}}\Delta t+\frac{1}{2}\ddot{x}\Delta t^{2\;\;\;\;}$$

(9) $$\dot{x_{k+1}}=\dot{x_{k}}+a\Delta t$$

(10) $${\vec{X}}_{k+1}=\left[\begin{array}{ccc} 1 & \Delta t & \frac{\Delta t^{2}}{2}\\ 0 & 1 & \Delta t\\ 0 & 0 & 1 \end{array}\right]{\vec{X}}_{k}\;\;\;\;\;\;;\;\;\vec{X}=\left[\;\begin{array}{ccc} \vec{x} & \vec{\dot{x}} & \vec{\ddot{x}} \end{array}\;\right]$$

where Δ*t* represents the average sampling time of the SVGS.

**PX4 Flight Controller**. The PX4 firmware provides a flight mode called “Offboard Mode”, where commands and setpoints can be sent to the FCU via the MAVLink protocol. This is used in the landing mission to send position setpoints via the MAVROS ROS package. In the Pixhawk-4′s offboard mode, the firmware provides a cascaded control architecture for the flight vehicle, with a combination of P controller for position, and a PID controller for velocity [24], as shown in Figure 10. In our experiments, the target’s position, velocity, and yaw estimates relative to the ground were obtained from the VIO sensor fusion with the Pixhawk internal IMU readings.

### 2.5. Coordinate Frames and Data Conversion

Two sources of position estimates are used: SVGS readings, which give the position of the target relative to the drone in a coordinate system attached to the camera; and the VIO sensor, which gives the position of the drone relative to a frame fixed to the ground. The VIO estimate is used by the FCU proprietary sensor fusion algorithm to generate a position estimate of the vehicle during flight relative to the ground. An outline of the PX4 VIO/IMU fusion logic to calculate position/attitude estimates is presented in Appendix A. The VIO sensor enables automatic flight in a GPS-denied environment: in the absence of a position estimate, the PX4 FCU will not enter automatic mode. The Intel T265 VIO sensor is more accurate than GPS, but would not be suitable for long-range or high-altitude maneuvers.

When using position estimates from the VIO sensor, the PX4 FCU operates in the FLU frame (Front–Left–Up). The origin of this frame is defined as the boot position of the vehicle, with the positive Front axis pointing to the front of the drone, and the positive Left axis pointing to the left of the drone. The orientation of the FLU frame is constant, regardless of the yaw angle of the drone: the FLU frame is always fixed to the ground and does not rotate with the drone.

SVGS position estimates from the Kalman filter (Equation (10)) need to be converted to the FLU frame, as shown in Figure 11. Since the FLU frame orientation remains the same regardless of the drone’s heading, the yaw angle from the VIO quaternion estimate is used in the SVGS frame rotation to convert SVGS readings to the drone’s FLU frame.

Once flight mode is switched to autonomous, SVGS readings are ignored if the vehicle’s velocity exceeds a maximum threshold, because SVGS sends void data packets if the target velocity exceeds the threshold (typically 7.5 cm/sec). When an SVGS reading that meets the velocity requirement is captured, it is vectorially added to the local position of the drone in the FLU frame at that moment, as shown in Figure 11. This becomes the target’s position estimate in the FLU frame, and is updated every time an acceptable SVGS reading is captured.

## 3. Results

The objectives are the demonstration and assessment of precision landing using SVGS. This includes: (i) precision landing to a static target using SVGS in a laboratory environment; (ii) precision landing with other state-of-the-art landing technology, such as augmented reality (AR) markers; and (iii) experimental comparisons to assess landing performance, using the Intel T265 VIO sensor as metrology framework. This required both software and hardware integration of the SVGS device with the flight vehicle (both the companion computer and FCU).

### 3.1. Speed Estimation from SVGS Measurements

A computer-controlled linear stage was used to assess the accuracy and performance of this estimator by providing known motion trajectories that can accurately be measured via the optical encoder in real time, and used to tune the corresponding Kalman filter parameters (Equations (8)–(10)). Using sinusoidal motion, position measurements from the linear stage and SVGS were recorded simultaneously while aligning the motion of the linear stage to the Z axis of SVGS, and the captured measurements were used to generate target velocity estimates. The results are shown in Figure 12, where the blue trace represents the true target velocity (from encoder measurements) and the orange trace represents the SVGS velocity estimate, obtained with the Kalman filter.

### 3.2. Results from Automated Landing Experiments

**Figure 7:**
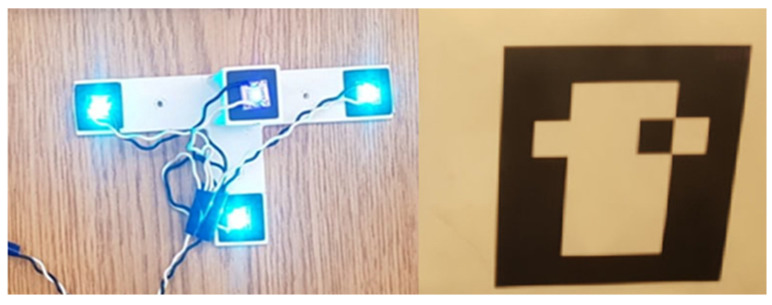
SVGS landing target (**left**), and ArUco landing target (**right**) [20,21].

**Figure 8:**
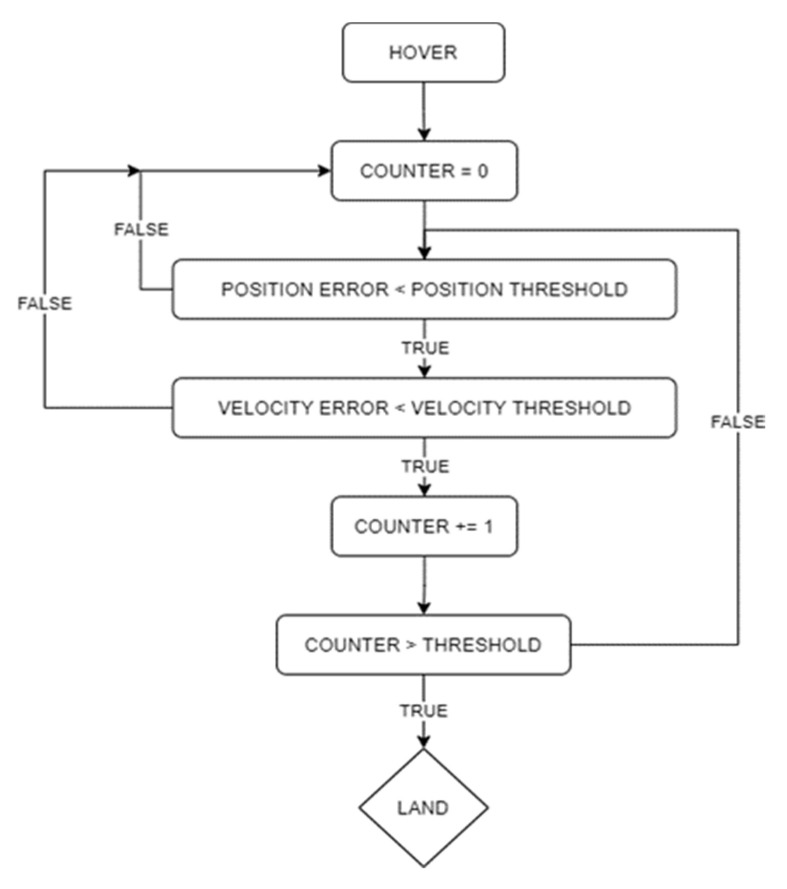
Autonomous landing loop flowchart.

**Figure 9:**
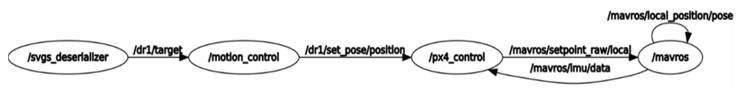
ROS nodes and topics for implementation of the automated landing maneuvers.

**Figure 10:**
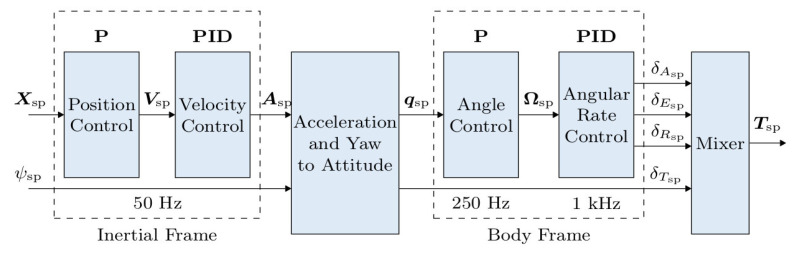
PX4 offboard multi-copter controller diagram [24].

**Figure 11:**
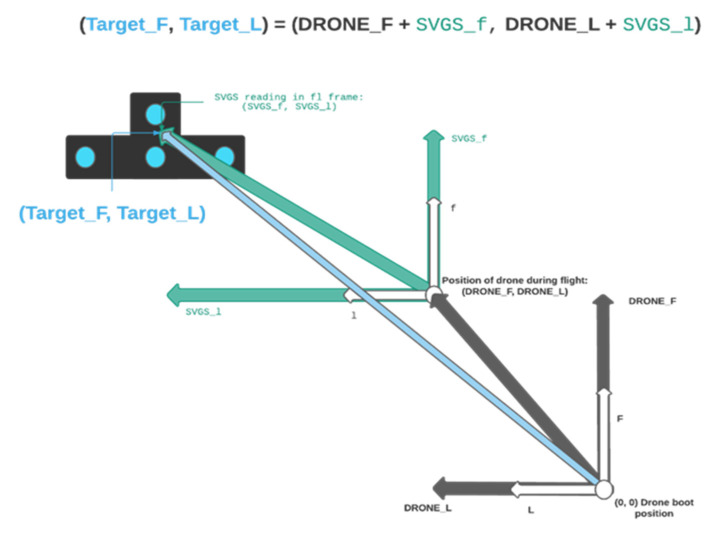
Estimation of the landing target position in the FLU coordinate frame.

**Figure 12:**
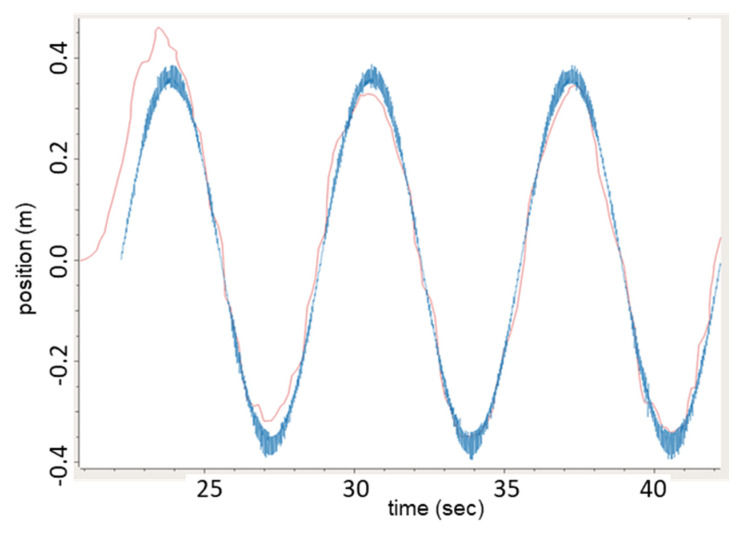
Kalman-based velocity estimation using the SVGS in sinusoidal motion experiments using ground-based linear stage. Orange: SVGS EKF velocity estimate; blue: linear stage true velocity.

Automated landing experiments followed the steps detailed in Section 2.3. For SVGS testing, the SVGS landing target was placed on the ground, powered by a four-cell battery connected to a voltage regulator to power the LEDs. For AR landing, a 10 × 10 cm marker (Figure 7) was placed on the ground. In both sets of experiments, the drone was piloted manually to the vicinity of the target; once a target was acquired in the camera field of view, autonomous mode was engaged. The functional flow diagram of the landing sequence is shown in Figure 8.

**Figure 13:**
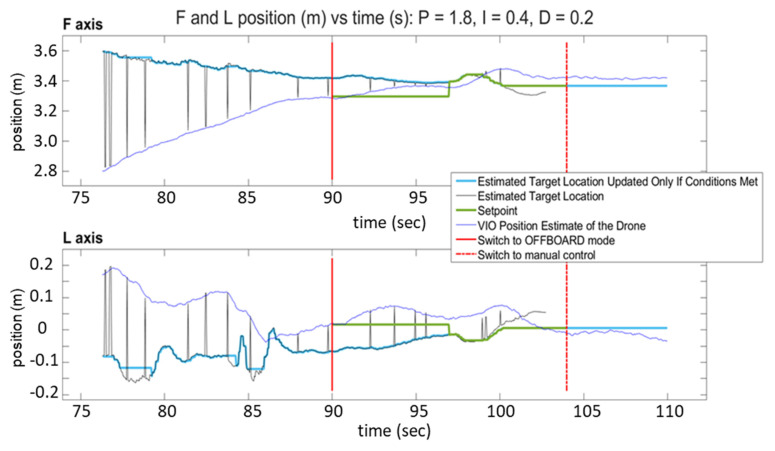
SVGS automated landing experiment 1: position vs. time for F and L axes.

**Figure 14:**
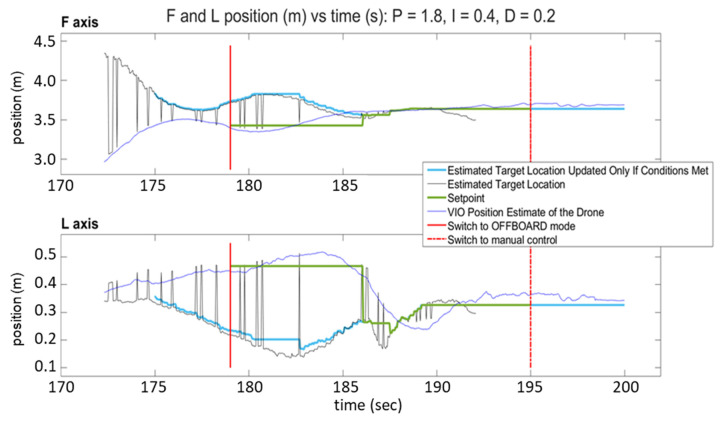
SVGS automated landing experiment 2: position vs. time for F and L axes.

Figure 13 and Figure 14 show the response of the quadcopter (position of the flight vehicle in the F and L axes) during two automated landing maneuvers using SVGS as a landing sensor with different PID settings. The plots show the part of the mission where the target is within the field of view of the sensor, and include two red vertical lines: a solid line to indicate transition to OFFBOARD mode when approaching the target, and a dash–dot line to indicate transition to manual control after landing.

The plots have four traces each: the VIO estimate of the drone’s position (blue), the target location estimated by SVGS (black), the landing position set point (green), and the estimated target location, updated only if drone velocity conditions are met (light blue). The black traces (SVGS estimate of target location) have spikes that reflect the fact that SVGS readings are discarded when the SVGS algorithm fails to converge. The position setpoint commands (green trace) are updated only after the hover period has concluded, the FCU operating mode is set to “OFFBOARD” mode, and a valid SVGS reading is taken within the maximum allowable velocity. Successful landing is demonstrated by the blue traces in both F and L axes converging to the position setpoint command, calculated based on SVGS readings. Figure 13 and Figure 14 show the results of two successful landing experiments for the same set of PID control parameters of the FCU’s OFFBOARD mode, for different approach velocities (0.15 m/s for Figure 13, and 0.25 m/s for Figure 14).

**Figure 15:**
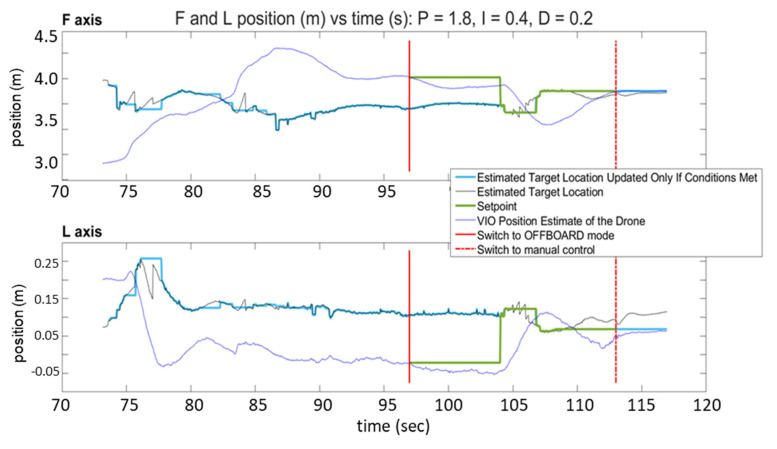
ArUCo automated landing experiment: position vs. time for F and L axes.

Figure 15 shows the position response of the vehicle in both F and L axes when landing using an ArUCo marker as the landing target. The color code of traces is the same as in Figure 13 and Figure 14. The target location is estimated with the Logitech webcam detecting the position and orientation of the ArUCo marker using OpenCV functions, and the position setpoint (green trace) is updated by the same logic as in the SVGS landing mission. Successful landing is again demonstrated by the blue traces in both F and L axes converging to the position setpoint command.

In the landing experiments, the prerequisite to start automatic landing was to reach target acquire of the landing beacon with SVGS before automatic motion control was engaged in the drone. This is caused by the narrow field of view of the smartphone camera at the approach altitude, and is therefore a common limitation in camera-based systems.

## 4. Discussion and Conclusions

Results from the landing experiments demonstrate the ability of SVGS to achieve successful automated landings by providing setpoint commands to the FCU in Offboard mode. The setpoint commands correspond to the target position estimated via the SVGS, converted to the FLU frame, and updated only if the vehicle’s magnitude of velocity vector is less than the acceptable threshold (7.5 cm/s).

Automated landing using SVGS and AR are similar techniques: both use the image processing of camera information to estimate the position and attitude of a landing target. In our experiments, the SVGS was able to capture target position from a farther distance during the approach compared with AR (1.34 m vs. 0.56 m). Landing performance for both SVGS and AR was similar (in terms of both accuracy and response time): for both technologies, the drone achieved automatic landing within the error margin assigned. AR requires good illumination conditions and a clear view of the landing target to succeed. SVGS has significant advantages in environments that are dusty, or when the illumination conditions are poor, because the landing beacon is self-illuminated (by four bright LEDs) and the intensity can be adjusted to better suit the local conditions. Furthermore, the dimensions of the SVGS beacon can be scaled to accommodate different operating ranges.

The computational load of the proposed method on the drone flight computer is minimal, since the SVGS algorithm runs on a separate unit (Android smartphone), and the landing logic is simple and runs in a powerful companion computer (Latte Panda Delta).

Assessment under various landing scenarios is needed to further demonstrate the performance of the proposed approach (e.g., robustness to noise) under different operational conditions. This can be implemented and tested by future users when adapting the proposed technique to the conditions of specific landing missions.

The use of an Android-based guidance system is a trade-off between added weight (and its effect on mission time and flight dynamics), versus the advantage of having the smartphone CPU handling the image processing. Vision-based guidance systems that use the onboard CPU for image processing are significantly affected by the weight and power consumption of the hardware required to handle image processing on board. The use of a smartphone represents a highly compact and lightweight solution to manage image processing, and does not affect the drone power or battery usage because it runs on its own battery. Other solutions that use different resources (CPUs and camera) to support SVGS deployment could be used, such as single-board Android development boards (e.g., by Inforce Computing) of GPU-based systems such as nVidia’s Jetson.

The SVGS app runs on Android, which introduces time uncertainty typical of any operating system (OS); this is a disadvantage when compared with a real-time sensor. The variability introduced by the OS in SVGS has been quantified and discussed in [14]. The update rate of MAVLink setpoints is 2 Hz, whereas the update rate of the SVGS is 10 Hz. The effect of the operating system time jitter in SVGS performance is negligible for update rates below 20 Hz. Therefore, in the operational conditions described in this paper, the effect of Android-induced time jitter can be ignored. In precision landing, the update rate of the vision system does not need to be high because the vehicle moves at low speed as it approaches the landing beacon—the use of a non-deterministic sensor therefore has a small impact on performance. Furthermore, the effect of the OS-induced time jitter on SVGS can be further compensated by the use of a Kalman filter, as shown in Section 3.1.

## Data Availability

Not applicable.

## Appendix A

This section presents an outline of the Kalman filter fusion algorithm of VIO and IMU measurements in the PX4 autopilot. First, the position estimate is corrected for the offset of the VIO sensor relative to the IMU. This is performed by subtracting the xyz position of the IMU in the body frame (imu_pos_body) from the xyz position of the VIO focal point in the body frame (ev_pos_body):

pos_offset_body = ev_pos_body − imu_pos_body (A1)

The offset is then converted into Earth coordinates using a transformation matrix from body to earth, R_to_earth:

Pos_offset_earth = R_to_earth * pos_offset_body (A2)

Position is then calculated by subtracting this offset from the position sample of the VIO:

pos = position_sample_VIO − pos_offset_earth (A3)

Position covariance is taken from the VIO (pos_cov). If GPS is used, pos_cov is the maximum between the VIO position covariance and the GPS position covariance:

(A4) $$\mathrm{pos}\_\mathrm{cov}=\max(\mathrm{pos}\_\mathrm{cov}\_\mathrm{vio},\;\sqrt{\mathrm{GPS}\;\mathrm{position}\;\mathrm{noise}})$$

Measurement is set to position:

measurement = pos (A5)

The measurement variance is set to the max between pos_cov, the position covariance of VIO, and √0.01 (the multiplier for a 99% confidence level):

(A6) $$\mathrm{measurement}\_\mathrm{var}=\max(\mathrm{pos}\_\mathrm{cov},\;\sqrt{\mathrm{VIO}\;\mathrm{position}\;\mathrm{noise}},\;\sqrt{0.01})$$

The observation is defined as:

observation = measurement − bias (A7)

The observation variance is set to the maximum between the addition of the measurement variance plus the bias variance, and √0.01:

(A8) $$\mathrm{obs}\_\mathrm{var}=\max(\mathrm{measurement}\_\mathrm{var}+\mathrm{bias}\_\mathrm{var},\;\sqrt{0.01})$$

The innovation is defined as the state minus the observation:

innovation = pos − observation (A9)

The variance in the innovation is set to the state covariance matrix, P, plus the observation variance:

innovation_variance = P + obs_var (A10)

The Kalman gain is then calculated as:

K = P/innovation_variance (A11)

The covariance corrections are applied, which leads to the updated position estimate:

KHP = K * P (A12)

P = P − KHP (A13)

pos = pos − K * innovation (A14)
